# The predictive value of hunger score on gastric evacuation after oral intake of carbohydrate solution

**DOI:** 10.1186/s12871-018-0470-3

**Published:** 2018-01-12

**Authors:** Qiu Weiji, Li Shitong, Luo Yu, Hua Tianfang, Kong Ning, Zhang Lina

**Affiliations:** 1 Department of Anesthesia and Critical Care Medicine, Shanghai Fourth People’s Hospital, 1878 North Sichuan Road, Hongkou District, Shanghai, 200081 China; 2 Department of Anesthesia, Shanghai First People’s Hospital affiliated to Shanghai Jiaotong University School of Medcine, 100 Haining Road, Hongkou District, Shanghai, 200080 China; 3 Department of Radiology, Shanghai Fourth People’s Hospital, 1878 North Sichuan Road, Hongkou District, Shanghai, 200081 China; 4 Department of Biostatistics, Shanghai Jiaotong University School of Medicine, 280 South Chongqing Road, Huangpu District, Shanghai, 200025 China

**Keywords:** Gastric emptying, Gastric residual volume, Magnetic resonance imaging, Numerical rating scale, Preoperative fasting, Subjective hunger feeling, ERAS

## Abstract

**Background:**

Surgical patients are asked to fast for a sufficient duration to ensure that the amount of residual liquid in the stomach is within the safe range, thereby reducing the risk of gastric reflux perioperatively. The authors hypothesized that subjective hunger numerical rating scale (NRS) score could also help assess the process of gastric emptying and determine the amount of fluid remaining in the stomach.

**Methods:**

The current study consisted of healthy volunteers recruited by advertisement and mutual introduction. Participants were asked to rate their subjective hunger feeling every 30 min after oral administration of 8 mL/kg carbohydrate nutrient solution that contained 10% maltodextrin and 2.5% sucrose. Consecutively, the gastric residual fluid was measured by magnetic resonance imagining (MRI). The Spearman’s correlation coefficient, the ROC curves and the stepwise regression were used to analyze the predictive value of NRS for the gastric emptying process.

**Results:**

The cohort consisted of 29 healthy volunteers enrolled in this study. The area under ROC curves estimated by the NRS score for the gastric residual volume of 2 mL/kg, 1 mL/kg, and 0.5 mL/kg were AUC_2.0_ = 0.78, AUC_1.0_ = 0.76, and AUC_0.5_ = 0.72, respectively. The correlation coefficient between the NRS score and the residual liquid in the stomach was −0.57 (*P* < 0.01). The correlation coefficient between the increase of the NRS score and the decrease of gastric liquid residual volume was 0.46 (*P* < 0.01). The standardized estimate of NRS score for the residual volume was −0.18 (*P* < 0.01) and the standardized estimate of fasting time was −0.73 (*P* < 0.01).

**Conclusions:**

The subjective hunger NRS score can not accurately predict the gastric residual volume, but it can provide a reference for clinicians to judge the gastric emptying process and it should be used as a second check after oral intake of clear fluids before surgery according to the new fasting protocol.

## Background

Preoperative fasting is a universally applied measurement to prevent patients, receiving anesthesia and surgery, from gastric reflux and aspiration pneumonia perioperatively. Although the recently amended guidelines invariably recommend shortening of the preoperative fasting duration and ingesting appropriate preoperative diet (water) to reduce the adverse effects of prolonged fasting on the human body [1–4], surgical patients are still required to fast for a sufficient period of time to ensure that gastric contents are drained to a safe range before anesthesia and surgery.

This is because gastric residual volume is considered as the most important predictor of the risk of gastric reflux episodes during anesthesia, while preoperative fasting is speculated as the most controllable parameter to ensure an empty stomach to prevent perioperative gastric reflux and inspiration pneumonia during anesthesia induction [5]. But we have already known from the literature that there is no guarantee the stomach could be emptied enough and the residual volume varies from person to person even after long time fasting [6, 7]. With the application of the enhanced recovery after surgery (ERAS) and new fasting protocol, surgical patients are encouraged to take preoperative fluids to reduce the uncomfortable feeling and hypovolumic status before surgery [8, 9]. The usage of simple and effective method to help determine the gastric residual volume after oral intake of the preoperative nutrient solution is yet inconclusive.

There are several studies on the subjective hunger feeling previously with different results [10–13]. Our study focused on the predictive value of subjective hunger numerical rating scale (NRS) score for the gastric residual volume and the gastric emptying process in adults, who can clearly express their feeling of hunger, especially after the ERAS and new fasting protocol have been applied. Our specific hypothesis was that subjective hunger NRS score would help the clinicians in a preliminarily finding on the gastric emptying process and predicting the gastric residual volume.

## Methods

### Materials

This prospective study was approved by the Ethics and Research Committee of the Shanghai Fourth People’s Hospital, (number 2015001, Shanghai China). Written informed consent was obtained from all the participants enrolled in the study.

Volunteers participated in the study were recruited from the local community by advertisement and mutual introduction. Inclusion criteria comprised of age between 18 and 70 y with American Society of Anesthesiologists’ physical status (ASA) class I to II. The exclusion criteria were abdominal surgery, gastrointestinal diseases, use of any drugs known to have an influence on the gastrointestinal motor function, metal stents and pacemakers implanted, claustrophobia, cognitive impairment, and those who were extremely depressive or anxious that could not collaborate.

### Pretreatment management

When the volunteers have confirmed the participation in the study, they were informed about the aim of the study and the whole procedure. They signed a written informed consent to participant in the protocol and conducted a self-rating depression scale (SDS) and self-rating anxiety scale (SAS), which would be reviewed by a psychiatrist to exclude the extremely depressive or anxious participants. The subjects were informed that they could withdraw from the study at any time if they did not want to continue the procedure. The participants were allowed to choose a date of convenience to go through the investigation procedure, and the investigators reminded them of the precautions 2 days before the study. All the participants were informed by a phone call to intake light diet and fast from 10 p.m. on the day before the study.

After the participants had arrived at the center at 8 a.m. on the day, the investigators measured their weight, height and vital signs, which would include heart rate, noninvasive blood pressure, and pulse oximetry to exclude the ill volunteers. The participants were also inquired about their daily habits with respect to breakfast. All the information was recorded.

### Carbohydrate nutritional solution

The nutritional solution was concocted by the hospital’s nutrition section on the study day, which contained 12.5% carbohydrate; the specific composition was 10 g maltodextrin and 2.5 g sucrose per 100 mL water. The solution was stored in a warm box with the constant temperature of 37 °C.

The participants were required to intake the carbohydrate nutritional solution as fast as they can within less than 3 min. Every participant was supplied with the solution according to their body weight, 8 mL/kg, equivalent to 1 g/kg carbohydrate of the body weight for every participant.

### Treatment protocol

The investigators performed an MRI measurement of the participants’ stomach to identify the basic residual volume after overnight fasting, and the time point would be marked as T_b_. Then, the participants would drink the prepared carbohydrate nutritional solution according to the requirements, and the time point was marked as T_0_. They were scheduled to undergo the MRI measurement of their stomach every 30 min after oral intake of the carbohydrate nutritional solution until 2 h, and the time points would be marked as T_30_, T_60_, T_90_, and T_120_ for the MRI scanning at 30, 60, 90, and 120 min, respectively.

In addition, the participants used the NRS score to rate the degree of their subjective hunger feeling at time points of T_0_, T_30_, T_60_, T_90_, and T_120_. The score was reported with an 11 point NRS, with 0 representing the feeling of least hungry, and 10 representing the feeling of worst hungry. They were asked to choose the numerical figure between 0 and 10, which could best describe their feeling of hunger and the investigators would record the rating score.

### MRI measurement

MRI has been used as a method to study the process of gastric emptying in recent years, which has been proved to have a high accuracy of up to 96%. And it has many other advantages, such as reproducible, no radiation, the measured data is more objective and the data can be repeatedly analyzed after being saved [14, 15]. So we chose it to measure the gastric emptying process in our study.

All the subjects underwent five serial epigastric MRI scans on a 1.5 T Siemens MR Avanto scanner with 8-channel body coil. The first serial scan was performed in the fasting state. The participants completed the other four serial epigastric MRI scans every 30 min after drinking the nutritional solution.

**Fig. 1:**
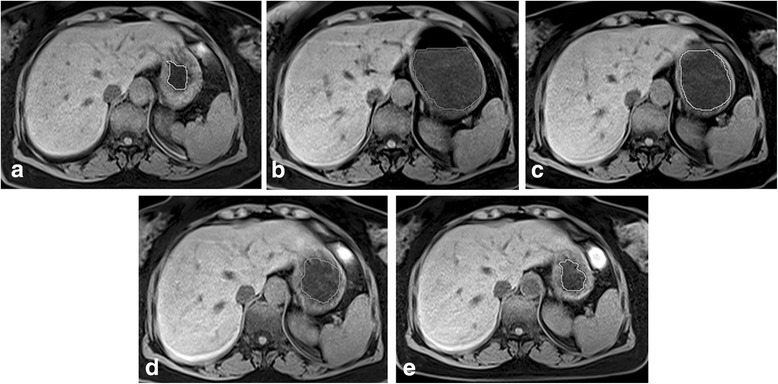
The participant underwent five serial epigastric MRI scans. The volume of fluid in the stomach was circled and calculated by AW suit software (GE company, USA) at the fasting state, 30 min, 60 min, 90 min, and 120 min after drinking carbohydrate solution separately (**a–e**)

All the participants were asked to hold their breath as long as they can during each MRI scanning protocol. Every MRI protocol included epigastric coronal T2_trufisp_2d [TR = 3.95 ms, TE = 1.71 ms, matrix = 384 × 512, 20 slices, slice thickness = 4 mm] and axial T1_vibe_3d [TR = 4.66 ms, TE = 2.15 ms, matrix = 195 × 320, 60 slices, slice thickness = 3 mm] (Fig. 1). The images were captured by a technician with 8 y of MR scan experience and reviewed by a radiologist to confirm whether the images encompassed the whole stomach. A 10-y-experienced radiologist input the T1WI-vibe imaging into AW suit software workstation (GE Company, USA) and calculated the volume of the residual liquid in the stomach three times. The mean value had been calculated as the final result.

### Statistical analysis

Data are presented as mean and SD for continuous variables. For all analyses, *P*-value <0.05 was considered statistically significant. SAS version 8.0 (SAS Institute, USA) was used for the analysis.

The relationship between the gastric residual volume measured by MRI and the subjective hunger NRS score at time points T_30_, T_60_, T_90_, and T_120_ was analyzed by Spearman’s correlation coefficient. With these data, we could also draw the receiver operating characteristic (ROC), and calculate the area under the curve (AUC), which would evaluate the predictive value of the NRS score for the gastric residual volume of 2, 1, and 0.5 mL/kg. Stepwise regression was used to analyze the predictive value of the NRS score and the fasting time on the gastric evacuation process.

## Results

29 healthy volunteers aged 26–66 y were enrolled in the study, including 14 males and 15 females, aged 58.6 ± 6.5 y, height 1.63 ± 0.08 m, body weight 66.4 ± 10.6 kg, BMI 20.9 ± 3.3 kg/m^2^. All volunteers completed the study as planned; no adverse reactions were reported.

At the time points of T_b_, T_30_, T_60_, T_90_, and T_120_, the residual liquid volumes (mL/kg) were 0.09 ± 0.06, 4.03 ± 0.87, 2.69 ± 0.96, 1.16 ± 0.67, 0.33 ± 0.28, respectively, while the subjective hunger NRS scores of the participants were 5 ± 1.79, 2.24 ± 1.52, 3.37 ± 1.61, 4.52 ± 1.81, and 5.74 ± 1.99, respectively (Table 1).

**Table 1:** Subjective Hunger NRS Score and Gastric Residual Volume (mL/kg)

	T_b_	T_30_	T_60_	T_90_	T_120_
Subjective Hunger NRS Score	5 ± 1.79	2.24 ± 1.52^*^	3.37 ± 1.61^*^	4.52 ± 1.81	5.74 ± 1.99
Gastric Residual Volume (mL/kg)	0.09 ± 0.06	4.03 ± 0.87^*^	2.69 ± 0.96^*^	1.16 ± 0.67^*^	0.33 ± 0.28^*^

^*^p < 0.05

**Fig. 2:**
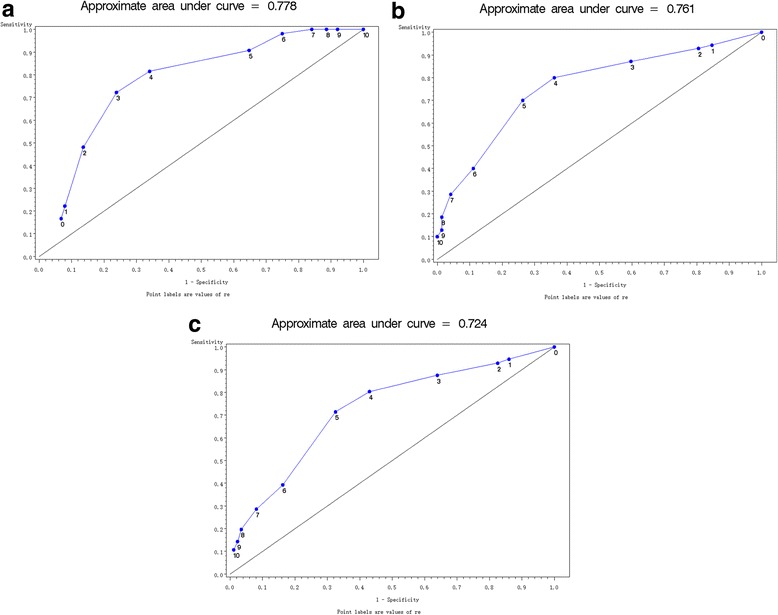
Area under the ROC Curve for the Gastric Residual Volume. **a** Area under the ROC Curve for Gastric Residual Volume (2 mL/kg), AUC_2.0_ = 0.78. **b** Area under the ROC Curve for Gastric Residual Volume (1 mL/kg), AUC_1.0_ = 0.76. **c** Area under the ROC Curve for Gastric Residual Volume (0.5 mL/kg), AUC_0.5_ = 0.72

The AUC with NRS predicting the gastric residual volume of 2, 1, and 0.5 mL/kg were AUC_2.0_ = 0.78, AUC_1.0_ = 0.76, AUC_0.5_ = 0.72 respectively, showing that the subjective hunger NRS score could not accurately predict the amount of fluid in the stomach (Fig. 2a-c).

The correlation coefficient between the NRS score and gastric liquid residual volume were −0.4 (*P* = 0.04), −0.4 (*P* = 0.03), −0.41 (*P* = 0.03) and −0.11 (*P* = 0.58) and the correlation coefficient between the increase of the NRS score and the decrease of gastric liquid residual volume were −0.06 (*P* = 0.82), −0.06 (*P* = 0.75), 0.13 (*P* = 0.5) and 0.09 (*P* = 0.67) at the time points of T_30_, T_60_, T_90_, and T_120_, separately, which showed a poor correlation. But when we calculate the correlation coefficients between them at all of the four time points together, they were −0.57 (*P* < 0.01) and 0.46 (*P* < 0.01) (Tables 2 and 3).

**Table 2:** Correlation Coefficient between NRS Score and Gastric Liquid Residual Volume

Time Point	T_30_	T_60_	T_90_	T_120_	TT
Correlation Coefficient	−0.4^*^	−0.4^*^	−0.41^*^	−0.11	−0.57^**^

^*^p < 0.05, ^**^p < 0.01

TT Calculation of all the four time points together

**Table 3:** Correlation Coefficient between Increase of NRS Score and Decrease of Gastric Liquid Residual Volume

Time Point	T_30_	T_60_	T_90_	T_120_	TT
Correlation Coefficient	−0.06	−0.06	0.13	0.09	0.46^**^

^**^p < 0.01

TT Calculation of all the four time points together

The stepwise regression was used to evaluate the prediction value of NRS score and fasting time on the gastric emptying process. The standardized estimate of NRS score was −0.18 (*P* < 0.01) and the standardized estimate of fasting time was −0.73 (*P* < 0.01).

## Discussion

The first finding of our study is that the subjective NRS score cannot accurately predict the amount of fluid remaining in the stomach after oral administration of the nutrient solution.

The intragastric fluid retention is considered as a vital index for assessing the risk of reflux episodes during anesthesia induction [1, 16]. Preoperative long duration of fasting has been applied to avoid gastric reflux; but we have already known that with long-time fasting, it could not fulfill the physiological requirements of the human body, nor is it necessary to fast for an extended duration to wait for the gastric residual volume to reach its safe area [17]. It has been reported that the gastric residual volume could be as much to cause gastric reflux even after a long time fasting [6, 7]. The recent guidelines for fasting time and methods have made similar amendments on the preoperative fasting, which suggest a shortening of the preoperative fasting time [1, 2] and appropriate diet or fluid to reduce the patients’ hunger feeling, preoperative discomfort, and tension [18, 19]. But the period of fasting is still the only crucial factor clinicians consider in judging if the patient’s stomach has been emptied to a safe range before the operation.

Hunger has an important role in the human body. It is a subjective feeling that would encourage people to consume food to meet their energy demands and maintain the body’s nutritional status and energy balance [10, 11, 20]. But the volume of food or fluid retained in the stomach and the accommodation of the stomach are not the only factors that could explain the feeling of satiety or hunger [10, 12, 20], which might be attributed to the complexity of this emotional experience. Previous studies have already shown that personal emotions, hormone levels, and the individual cognitive ability of hunger, as well as, the food tastes or the surrounding ambient temperature may affect the individual feelings of hunger [11, 21–25]. In other words, the same hunger NRS score might indicate a different amount of residual in the stomach with individual differences; while each person could have a different hunger rating score for the same amount of residual in the stomach.

When we calculated the AUC of 2, 1, and 0.5 mL/kg of the residual gastric liquid with the subjective hunger NRS score of the volunteers, we got AUC_2.0_ = 0.78, AUC_1.0_ = 0.76, and AUC_0.5_ = 0.72 (Fig. 2a-c). When we calculated the correlation coefficient between the NRS score and gastric liquid residual volume, and the correlation coefficient between the change of the NRS score and the change of gastric liquid residual volume at the time points of T_30_, T_60_, T_90_, and T_120_, separately, it showed a poor correlation between them (Tables 2 and 3).

In conclusion from these findings, the NRS score for subjective hunger does not have an optimal value for estimating the accurate amount of fluid remaining in the stomach. This finding was in accordance with several previous studies [10–13].

Another major finding of our study was the correlation between the NRS score and gastric emptying, whereby NRS was considered useful for the preoperative assessment of gastric emptying in addition to fasting time.

When we calculated all the four-time points of T_30_, T_60_, T_90_, and T_120_ together, we incorporated more research samples for statistics. The correlation coefficient between the NRS score and the gastric liquid residual volume was −0.57 (*P* < 0.01) and that between the increase of the NRS score and the decrease of gastric liquid residual volume was 0.46 (*P* < 0.01). We also used stepwise regression to calculate the standardized estimate of NRS score and the standardized estimate of fasting time for the prediction of gastric residual volume, they were −0.18 (*P* < 0.01) and −0.73 (*P* < 0.01). We speculate from these findings that the subjective hunger NRS score could help the clinicians with preliminarily finding on the gastric emptying process.

The period of fasting time has been the only criterion assessing whether the stomach is emptied enough before surgery for decades [26], but there is still significant difference of the gastric residual volume between the individuals with the overnight fasting gastric volume of up to 200 ml can be found [6, 7]. Nowadays the concept of ERAS encourages clinicians to use multimodal interventions to reduce the undesirable sequelae of surgical injury with improved recovery and reduction in postoperative morbidity and overall costs [8, 9], and one of them is shortening of the preoperative fasting duration and ingesting appropriate preoperative diet (water) before surgery [1–4]. We designed this study in an attempt to find a simple and noninvasive way to help the clinical anesthesiologists to make a rapid judgment on the gastric emptying process and gastric liquid residual volume, especially after oral administration of nutrient solution. We recruited healthy volunteers to simulate the newly recommended fasting protocol before surgery and asked them to take clear fluids to measure the emptying process according to the new fasting protocol. Previous studies have used gastric tube aspiration, scintigraphy, dilution of the solution, CT scanning, ultrasound and MRI measurements [7, 15, 16, 27–29] to measure the gastric residual volume. Owing to the complexity of the operation of these methods or that increasing the patients’ discomfort before surgery, none of these methods have been considered to be applicable for the preoperative assessment of residual liquid in the stomach.

The period of fasting time is still widely used throughout the world and has remained as somewhat the only criteria to make sure that the stomach is emptied before surgery, even though the literature and the reality have told us that even after a long period of fasting, there would still be some people whose stomach contains residuals enough to cause gastric reflux [6, 7]. The period of fasting time could not always be a perfect parameter to determine if the stomach has been emptied. From our findings of this correlation between the NRS score and the gastric liquid residual volume and the standardized estimate of NRS score for the gastric residual volume, we suggest the subjective hunger NRS score and the change of it be used as a second check for elective patients. And we believe that to ask the patients if they feel hungry and to rate the extent of their hunger in addition to asking about the period of fasting time at the same time, would be able to help the clinicians in a preliminarily finding on the gastric emptying, and help them better judge the stomach emptying process and the risk of gastric reflux than to use the traditional period of fasting time as the only indicator, especially after oral intake of the nutritional solution before surgery.

At last, our results showed that the NRS scores of T_30_ and T_60_ were significantly lower than the T_b_ point and increased gradually after oral administration of the nutrient solution, which was consistent with the trend of gastric emptying (Table 1). The liquid meal was concocted with a 12.5% carbohydrate nutritional solution, with a specific composition of 10 g maltodextrin and 2.5 g sucrose per 100 mL water. At the time point of T_120_, the stomach was almost emptied from our observation, and the average calorie emptying rate for the total 120 min was 2.2 kcal/min, which is in accordance with several previous reports [30, 31]. We speculate from these findings that the oral intake of the 12.5% carbohydrate nutritional solution can play a role in alleviating the hunger feeling of the participants to a certain degree, and the stomach could be emptied enough after the oral administration before 2 h in our volunteers.

Nevertheless, there are multiple limitations to our study. We find the correlation between the gastric residual volume and the NRS score, but the predictive value of the NRS score for the exact volume of the residual volume was moderate, which might be attributed to the significant intra- and inter-individual variation in NRS score and it could explain some of the previous findings [10–13]. The NRS score is worth further investigation among different ethnic groups [32] and in different preoperative fasting circumstances. We excluded the extremely anxious and depressive ones to avoid the over-emotional participants who might not collaborate well during the study. The preoperative patients were found in an anxious state, and thus, our study does not reflect this population. Although previous studies have shown that the psychological state does not affect the evacuation process [33, 34], we have planned to include such participants in the future study. In the current survey, we monitored the volunteers who were clear and could express their feeling clearly. When a critically ill patient is undergoing surgery, he/she could not accurately describe the hunger feeling, and thus, we would not be able to estimate the gastric emptying process with the NRS score.

Despite these limitations, our study describes the relationship between the subjective hunger NRS score and gastric residual volume after oral administration of carbohydrate nutrient solution. When the period of fasting time is still one of the most important factors that could help determine the gastric residual volume, our study describes the correlation between the subjective NRS score and the gastric residual volume. We conclude from these findings that our body can feel our own hunger, and this feeling of hunger expressed in NRS score could be used as a second check before surgery in addition to the traditional inquiry about the period of fasting time, which might help determine the emptying process of the stomach.

## Conclusions

In summary, the oral administration of 12.5% carbohydrate solution 2 h before can alleviate the hunger feeling among fasting volunteers for a certain period of time. Our finding is consistent with several previous findings that the subjective hunger NRS score may not be a good tool to accurately predict gastric volume. But the NRS score of subjective hunger feeling and its change were correlated with the amount of gastric fluid remaining after oral administration of the nutrient solution, which could provide a reference for clinicians to predict the gastric emptying process, especially after oral administration of the nutrient solution, and it could be applied as a preliminarily check after oral intake of clear fluids before surgery according to the ERAS and new fasting protocol.

## Abbreviations

ASA: Anesthesiologists’ physical status
AUC: Area under the curve
MRI: Magnetic resonance imagining
NRS: Numerical rating scale
ROC: Receiver operating characteristic
SAS: Self-rating anxiety scale
SDS: Self-rating depression scale
